# Early use of high-efficacy therapies in multiple sclerosis in the United States: benefits, barriers, and strategies for encouraging adoption

**DOI:** 10.1007/s00415-024-12305-4

**Published:** 2024-04-14

**Authors:** Barry A. Singer, Jenny Feng, Horacio Chiong-Rivero

**Affiliations:** 1 The MS Center for Innovations in Care, Missouri Baptist Medical Center, St Louis, MO USA; 2 Ochsner Medical Center, New Orleans, LA USA; 3 John Peter Smith Hospital, Fort Worth, TX USA

**Keywords:** Multiple sclerosis, High-efficacy therapies, Disease-modifying therapies, United States, Shared decision-making, Health care professionals

## Abstract

Multiple sclerosis (MS) is characterized by progressive neuroinflammation and neurodegeneration from disease onset that, if left untreated, can result in the accumulation of irreversible neurological disability. Early intervention with high-efficacy therapies (HETs) is increasingly recognized as the best strategy to delay or mitigate disease progression from the earliest stages of the disease and to prevent long-term neurodegeneration. Although there is growing clinical and real-world evidence supporting early HET intervention, foregoing this strategy in favor of a traditional escalation approach prioritizing lower-efficacy disease-modifying therapies remains a common approach in clinical practice. This review explores potential health care professional- and patient-related barriers to the early use of HETs in patients with MS in the United States. Barriers can include regulatory and reimbursement restrictions; knowledge gaps and long-term safety concerns among health care professionals; and various individual, cultural, and societal factors affecting patients. Potential strategies for overcoming these barriers and encouraging early HET use are proposed.

## Introduction

Multiple sclerosis (MS) is a chronic, immune-mediated neuroinflammatory and neurodegenerative disease of the central nervous system (CNS) [1]. MS is traditionally categorized into clinically isolated syndrome, relapsing–remitting MS (RRMS), primary progressive MS (PPMS), and secondary progressive MS (SPMS) [1], but there is increasing evidence that these subtypes form a continuous spectrum, with blurred boundaries between relapsing and progressive subtypes [2, 3]. MS disease activity and underlying neuroinflammation activity are typically indicated through clinical relapses, magnetic resonance imaging (MRI) activity, and worsening disability [4].

Because inflammatory disease activity and irreversible neurodegeneration can occur early in the disease course, even before the first clinical event [5, 6], early therapeutic intervention is recognized as giving the best long-term prognosis, compared with delayed or no intervention [7–9]. Despite this, many people with MS do not receive appropriate timely treatment, which can lead to poor disability outcomes [3, 10]. Selecting the optimal disease-modifying therapy (DMT) for newly diagnosed patients with MS is complex because our ability to precisely predict the MS disease course is limited. This is further complicated by drug-specific factors, such as mechanism of action, route of administration, dosing schedule, efficacy, and safety profile [11, 12]. Real-world evidence is emerging that early intervention with high-efficacy therapies (HETs) may provide the best opportunity to protect the CNS against irreversible injury and substantially mitigate the early inflammatory component of disease [13–17]. Until ongoing randomized controlled trials are able to confirm this stance from the clinical perspective [18, 19], there remains no formal consensus on the best therapeutic approach to MS care. Accordingly, those who may benefit from receiving HETs early in the disease course are likely to face more barriers to HET access than with traditional DMTs. These include restrictions imposed by reimbursement bodies; access to MS specialist neurologists and clinics; and patient-specific considerations, including socioeconomic background, ethnicity, geographic location, health insurance status, physical disability, comorbidities, long-term safety concerns, and family planning [7].

This review explores the obstacles to an early HET intervention strategy as related to health care professionals (HCPs) and patients with MS in the United States (US). The rationale and challenges of early HET adoption from the European perspective have been discussed elsewhere [10]. We also discuss potential strategies for overcoming these challenges and improving care for people with MS in the US who are underserved.

## Classification of DMTs by efficacy

For the purposes of this review and in lieu of comprehensive comparative trials, we have classified DMTs by efficacy into three categories based on their ability to reduce annualized relapse rates (ARRs) in phase 3 clinical trials. ARR was the primary outcome in a majority of these trials and has been used in similar DMT categorization approaches [20–23]. Low-efficacy therapies reduce ARR by 20–40% vs placebo in clinical trials and comprise the first-generation injectables interferon (IFN) beta and glatiramer acetate, and the second-generation oral medication teriflunomide (Table 1). Moderate-efficacy therapies reduce ARR by 40–60% vs placebo or by < 50% vs active comparator in clinical trials and include fumarates, cladribine, and the sphingosine-1-phosphate (S1P) receptor modulators (fingolimod, siponimod, ozanimod, and ponesimod). We have defined HETs as those that have reduced ARR by ~ 50% or more vs active comparator or by > 65% vs placebo in clinical trials; these include the US Food and Drug Administration–approved monoclonal antibodies for use in MS: natalizumab, alemtuzumab, ocrelizumab, ofatumumab, and ublituximab [8, 22, 24–26]. Other meta-analyses have similarly ranked monoclonal antibody DMTs, oral therapies (except teriflunomide), and then glatiramer acetate, interferons, and teriflunomide in descending order of efficacy based on their ARR vs placebo [22, 23].

**Table 1:** Efficacy of DMTs for MS according to reduction in ARR relative to active comparator or placebo, as determined from the approved dose in pivotal clinical trials

	DMT, class	Comparator	Clinical trial	Reduction of ARR
High efficacy^a^	Anti-CD20 monoclonal antibody			
	Ocrelizumab [27]	IFN beta-1a SC 44 mg	OPERA I/II	46–47%
	Ofatumumab [28]	Teriflunomide 14 mg	ASCLEPIOS I/II	50–60%
	Ublituximab [29]	Teriflunomide 14 mg	ULTIMATE I/II	50–58%
	Anti-CD52 monoclonal antibody			
	Alemtuzumab [30, 31]	IFN beta-1a SC 44 mg	CARE-MS I/II	49–55%
	Anti-a4 integrin receptor monoclonal antibody			
	Natalizumab [32]	Placebo	AFFIRM	68%
Moderate efficacy^b^	S1P receptor modulators			
	Fingolimod [33, 34]	Placebo	FREEDOMS I/II	48–54%
	Siponimod [35]	Placebo	EXPAND	55%
	Ozanimod [36, 37]	IFN beta-1a IM 30 mg	RADIANCE/ SUNBEAM	21–48%
	Ponesimod [38]	Teriflunomide 14 mg	OPTIMUM	31%
	Purine analog			
	Cladribine [39]	Placebo	CLARITY	58%
	Fumarates			
	Dimethyl fumarate [40, 41]	Placebo	CONFIRM/DEFINE	44–53%
	Diroximel fumarate [42]		EVOLVE-MS-1	
Low efficacy^c^	Pyrimidine synthesis inhibitor			
	Teriflunomide [43, 44]	Placebo	TOWER/TEMSO	31.5–36%
	Amino acid copolymer			
	Glatiramer acetate [40, 45]	Placebo	CONFIRM/GALA	29–34%
	IFNs			
	IFN beta-1a [46]	Placebo	PRISMS	33%

*ARR* annualized relapse rate, *DMT* disease-modifying therapy, HET high-efficacy therapy, *IFN* interferon, *IM* intramuscular, *MS* multiple sclerosis, *S1P s*phingosine-1-phosphate, *SC* subcutaneous

^a^HETs reduce the ARR by ~ 50% or more vs active comparator or > 65% vs placebo

^b^Moderate-efficacy therapies reduce the ARR by 40–60% vs placebo or < 50% vs active comparator

^c^Low-efficacy therapies reduce the ARR by 20–40% vs placebo

An important note is that this classification based on ARR does not account for DMT effects on MRI, disability, or other disease measures, and certain DMTs might be more effective on measures other than ARR. For instance, S1P modulators and cladribine are considered moderate-efficacy DMTs according to the above definition and non-HETs in other publications yet have also been defined as HETs elsewhere [9, 13–15, 47]. Categorizing higher-efficacy DMTs using data from active comparator studies can therefore enable more granulated delineation between high and moderate efficacy.

## Existing treatment paradigms

An escalation strategy is one in which patients initially receive lower-efficacy therapies with well-characterized safety profiles to minimize concerns about side effects [48, 49]. HCPs then switch patients to higher-efficacy therapies following breakthrough disease activity (i.e., new clinical relapses and/or MRI activity) [48, 49].

Initial intervention with lower-efficacy therapies might benefit some patients, such as those with a mild disease course or a preference for lower-risk DMTs [50]. Escalation might also allow some patients flexibility with treatment options, taking into account prognostic measures, risk tolerance, age, and available financial resources [24]. Because lower-efficacy DMTs are thought to be more immunomodulatory than immunosuppressive [51], they can be particularly useful for patients with comorbidities, such as chronic infections, where immunosurveillance is essential. The clinical data suggest that low-efficacy therapies may have value in reducing inflammation, mild relapses, and disability progression in the beginning of the disease; however, their effect on accumulation of disability may not be maintained long-term [52–54].

Society guidelines have traditionally recommended an escalation approach [55, 56]. Patient care is now evolving, with many MS specialists and other clinicians prioritizing treatment goals of mitigating or halting the underlying inflammatory mechanisms of MS to prevent irreversible disability [8, 47]. This has led to a shift toward an early aggressive or proactive treatment approach, where patients are initiated on HETs (often at diagnosis) to more effectively prevent relapses, reduce potential neuronal injury, slow disability accrual, and ultimately improve optimal patient outcomes [8, 48].

### Efficacy and safety of lower-efficacy therapies

Clinical trials of patients with RRMS have revealed comparable reductions in ARR for IFNs, glatiramer acetate, and teriflunomide and largely similar effects on time to first relapse for IFNs and glatiramer acetate (Table 1) [40, 43–46, 57, 58]. Both teriflunomide and IFN therapy have been reported to delay disability progression [43, 46, 59, 60], whereas glatiramer acetate appears to have no significant impact other than a potential stabilizing effect of long-term disability progression in patients with mild disease activity [45, 53].

In terms of their safety profiles, IFNs and glatiramer acetate are generally well tolerated and are most commonly associated with injection-site reactions [11]. Both are considered safe for use during pregnancy, have a perceived lower risk of infections compared with higher-efficacy therapies, and only minimally affect immune responses to vaccines [26, 61]. Common adverse events (AEs) with teriflunomide include hypertension, diarrhea, and hair loss [59, 62]. Regular liver function testing and use of effective contraception is advised for teriflunomide as it carries a black box warning for severe liver injury and teratogenicity, although recent post-marketing pregnancy registry data suggest variable outcomes [62–65]. Teriflunomide is eliminated slowly from the plasma, and a wash-out with cholestyramine prior to switching to another DMT is advisable because it can remain in the blood for up to 2 years after the last dose [62].

In patients with RRMS, S1P modulators, dimethyl fumarate, and cladribine led to greater reductions in ARR vs low-efficacy therapies or placebo (Table 1) [36–41, 66, 67]. Although these moderate-efficacy therapies are mostly well tolerated, some notable safety considerations include warnings of malignancy and teratogenicity with cladribine [68]; gastrointestinal AEs with dimethyl fumarate [40]; and an increased risk of cardiovascular events, macular edema, and rare serious opportunistic infections, such as progressive multifocal leukoencephalopathy (PML) and cryptococcal meningitis with S1P receptor modulators [69–72].

### The need for effective therapies in early MS

Disability in MS is one of the main drivers of poor quality of life among people with MS [73–76]. Disability accrual occurs primarily via two mechanisms: relapse-associated worsening (RAW) due to acute lesions and incomplete recovery from relapses and progression independent of relapse activity (PIRA) encompassing the gradual clinical progression from disease onset [77]. RAW is a form of acute neuroinflammation that appears to be driven by peripherally activated B cells and T cells, whereas PIRA (also known as “smoldering inflammation”) can be primarily attributed to pathogenic microglia in the CNS that drive progressive neuroaxonal loss [78, 79]. Meningeal lymphoid aggregates and subpial cortical lesions contribute to neurodegeneration [80]. Chronically active MS lesions (including paramagnetic rim lesions), characterized by macrophage-mediated injury, contribute to smoldering inflammation and potentially PIRA pathogenesis [78, 80]. PIRA plays a significant role in disease worsening and, over time, may become the main contributor to disability progression [77, 81]. Because current DMTs are limited in their abilities to stop PIRA, early and effective intervention may therefore be the optimal strategy to prevent or delay long-term irreversible disability.

Two ongoing prospective, randomized pragmatic trials (TREAT-MS [NCT03500328] and DELIVER-MS [NCT03535298] [18, 19]) are directly comparing early HET vs escalation therapy in terms of disability progression, relapses, neurodegeneration, health-related quality of life, burden of MS, cognition, employment, and safety. Data from these trials will shed more light on the wider benefit of early HET to the patient, beyond clinical efficacy and safety measures.

## Benefits and safety considerations of early HET

### Efficacy and risk of disability progression

The question of whether early HET or escalation therapy delivers the best long-term outcomes for patients with MS hinges on conclusive evidence from randomized controlled trials for there to be any consensus among MS neurologists. While DELIVER-MS and TREAT-MS aim to definitively answer this question, some extrapolations can be made from pivotal comparator-controlled trials of HETs in treatment-naïve patients. In the CARE-MS I trial of patients with early RRMS, alemtuzumab led to reduced relapse rates, slower brain volume loss, and more patients remaining free from clinical disease activity compared with IFN beta-1a [30]. Similarly, natalizumab substantially reduced the risk of disability progression and relapse rate in patients with highly active RMS in the AFFIRM and SENTINEL trials [82]. The OPERA I/II and ORATORIO clinical trials of ocrelizumab enrolled patients with RMS and PPMS, respectively, most of whom had not received DMTs in the previous 2 years. Both trials demonstrated improved efficacy outcomes (ARR, disability progression, lesion burden, physical and cognitive scores) vs IFN beta or placebo [27, 83]. In recently diagnosed patients with relapsing MS from the ASCLEPIOS I/II clinical trials, ofatumumab led to significantly reduced ARR, fewer MRI lesions, and increased odds of achieving “no evidence of disease activity” vs teriflunomide [84]. In the ULTIMATE I and II clinical trials, patients with RMS treated with ublituximab experienced a lower ARR and fewer brain lesions on MRI than with teriflunomide but no significantly lower risk of disability progression [29]. The effect of HET on relapse rates in clinical trials implies protection against RAW, but what about PIRA? In ASCLEPIOS I/II, ofatumumab significantly reduced the risk of experiencing PIRA events over a 6-month period compared with teriflunomide [84]. Likewise, OPERA I/II found ocrelizumab to be superior to IFN beta-1a in preventing PIRA over 12 and 24 weeks [81]. However, the question of whether mitigating early PIRA events can prevent long-term neurodegeneration is beyond the scope of these trials.

Real-world evidence has revealed clinical benefits, lower disease progression, and favorable long-term outcomes for patients receiving early HET vs escalation therapy or delayed HET [13, 15, 85–87]. In an observational study using data from the MSBase and Swedish MS registries, HET treatment initiated within 2 years of disease onset reduced the risk of disability progression by 66% after 6–10 years compared with HET initiated later in the disease course (hazard ratio 0.34; 95% confidence interval, 0.23, 0.51) [14]. Two similar studies comparing data from Danish, Czech, and Swedish MS registries found that the Swedish high-efficacy induction strategy resulted in reduced risk of disability progression and relapses compared with the Danish and Czech escalation strategies [16, 88]. Further, a systematic review of seven studies revealed that early HET had a 30% reduction in disability worsening at 5 years vs escalation therapy [89]. It should be noted that because some real-world studies include S1P modulators in their definition of high efficacy [16, 85, 86]; the true beneficial effects of HETs as characterized in this review may be underestimated depending on the respective HET definitions of each study.

Patients who received HET on the basis of more active disease actually had a lower long-term risk of conversion to SPMS than those with less active disease on escalation therapy [85]. Even after patients switched from moderate-efficacy therapy to HET, those who initiated early HET showed improved longer-term outcomes compared with those on delayed HET [14, 86]. Younger age is a major factor of immunomodulatory drug efficacy [90], likely due to higher cerebral reserve, further reinforcing the importance of early treatment. Overall, the available data indicate that patients can achieve maximum therapeutic benefit with early HET therapy, regardless of prognostic factors or disease severity [10, 91].

### Safety and risk of AEs

Patients receiving HETs do not necessarily experience more AEs than those receiving lower-efficacy therapies. In an observational study of 4861 patients, the proportion of patients discontinuing treatment due to AEs was comparable between those on an early HET strategy (where one-third of patients received primarily first-line rituximab or natalizumab) and those on an escalation strategy (where nearly all patients received first-line low- or moderate-efficacy therapy, mostly teriflunomide) [16]. Similarly, a systematic review of two studies reported a similar safety profile between early HET and escalation strategies, with comparable proportions of serious AEs [89]. The OPERA, ASCLEPIOS, and ULTIMATE trials further showed that, aside from infusion- or injection-related reactions, the safety profiles of ocrelizumab, ofatumumab, and ublituximab were generally similar to the lower-efficacy therapies IFN beta and teriflunomide [27, 29, 84]. Low serum immunoglobulin (Ig) levels can occur with lymphocyte-depleting HETs and have been associated with increased infection risk [92–94]. However, in patients treated with ofatumumab for up to 5 years, mean immunoglobulin (Ig)G levels remained stable and mean IgM levels decreased but remained above the lower limit of normal [95]. Although no link between reduced Ig levels and risk of serious infection was found [95], this potential risk can nonetheless be mitigated by careful laboratory and clinical monitoring [26].

Longer-term safety analyses of HETs have been reported, although this is currently limited for newer HETs, such as ublituximab. Infusion-related reactions, opportunistic infections, and serious infections remain among the most frequently reported AEs [96–99]. Natalizumab treatment has been associated with increased incidence of PML (estimated to occur at rates of 0.01–10 per 1000 individuals with John Cunningham virus positivity) with increasing risk depending on the duration of treatment (especially over 2 years) and prior immunosuppressant therapy [99–101]. Safety data for ofatumumab for ≤ 3.5 years or ocrelizumab for ≤ 5 years have not shown increases in the incidence and risks of AEs over those reported in the clinical trials [97, 98]. Alemtuzumab is associated with secondary autoimmune disease (particularly thyroid disorders) that can occur post treatment with delayed onset, although the risk decreases in the fourth year after the last dose [96, 102]. Serious safety considerations for alemtuzumab include the risk of infusion-related ischemic and hemorrhagic stroke and cervicocephalic arterial dissection [103]. Longer-term safety analyses indicate that the incidence of AEs, such as infusion-related reactions and infections, reduce over time with ongoing HET treatment [96, 97, 99, 102]. Careful monitoring, patient education, and risk mitigation can facilitate early detection and effective management of AEs [96, 104]. Taken together, the emerging long-term data on HETs suggest that these therapies should not be excluded solely on the basis of their safety profiles, but consideration of individual patient factors is warranted. There remains an unmet need for robust long-term data comparing the safety of higher- and lower-efficacy therapies to fully address perceived safety risks with HET treatment.

### Socioeconomic benefits of early treatment

MS-related disability progression can have deleterious effects on society and the economy. Disability is a chief driver of costs, which greatly increase as disability level increases [76, 105]. Dependency on medication and health care resources, in addition to increasing usage of informal care, substantially contribute to these rising expenses [105]. The indirect costs of care and loss of productivity of patients and their caregivers are responsible for the greatest financial burden in MS [106]. The largest contributors to these costs are lost earnings due to presenteeism (defined as presence at work without productivity), absenteeism, and premature death [107]. The MS Cost of Illness Study found that the probability of working, work hours, and work productivity all reduced with increasing subjective cognitive impairment and fatigue [108]. In particular, MS-related fatigue is a highly prevalent symptom in clinical practice and affects approximately 80% of people with MS [109–111], although figures of > 90% have been reported [111]. In people with MS, presenteeism and absenteeism have been linked to physical and cognitive fatigue, symptom severity, depression, anxiety, and disability [112, 113].

Cost-effective analyses of early vs delayed initiation of HET in MS confirm a positive socioeconomic impact of early intervention. The lower overall costs from reducing disability with early HET can compensate for the initial expense of the medications [114, 115]. Lower incidence of relapses and delaying or preventing disease progression could lead to a decrease in health care resource utilization and associated costs [116–118]. In fact, early HET can be more cost-effective than an escalation approach at reducing disability progression within a 5-year period [89]. As a result of improving patient health, effective therapeutic intervention from diagnosis could mitigate the societal and economic burden of disease.

## Barriers to adoption of early HETs and potential solutions

**Fig. 1:**
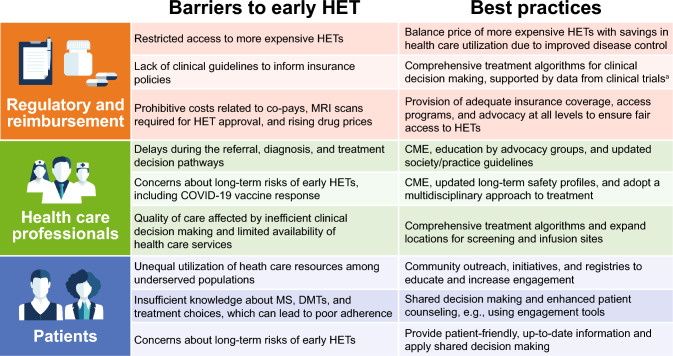
Proposed best practices for overcoming barriers to early high-efficacy therapy (HET) treatment in multiple sclerosis (MS) from the perspective of regulatory and reimbursement agencies, health care professionals, and patients. ^a^Anticipated clinical trial data from DELIVER-MS and TREAT-MS to support the benefit of early HET treatment in MS outcomes. *CME* continuing medical education, *COVID-19* coronavirus disease 2019, *DMT* disease-modifying therapy, *MRI* magnetic resonance imaging

Analyses of US treatment patterns including data up to 2020 have revealed a rising number of HET initiations over time [119, 120], an upward trend that market growth forecasts indicate is likely to continue. Despite this, moderate-efficacy oral therapies were initiated most often up to 2020, by a substantial margin [120]. More up-to-date reports on current HET usage are limited in part due to the delayed availability of real-world data capturing more recently approved HETs (i.e., ofatumumab, ublituximab). Nonetheless, while it can be assumed that even more patients in the US will receive HET treatment in the coming years, difficulties in accessing this treatment are likely to remain for the individual. Social and cultural factors affecting access to care, insurance company requirements, financial burdens, and perceptions of risk are just a few well-documented examples [121–123]. The issue is further compounded by a lack of clear guidelines on initiating HET early in the disease course that could preclude many first-line treatment options. The potential challenges and proposed solutions to strengthen the early HET approach are outlined in the following sections and summarized in Fig. 1.

### Regulatory and reimbursement factors

Conflicting priorities between insurance companies and patients is a significant contributor to disparities in access to MS treatments. US insurance companies can restrict or deny access to some DMTs (despite their regulatory approval) and often insist that lower-efficacy therapies be used first [124, 125]. High rates of medication denials occur in both commercial and government (i.e., Medicaid) insurance programs and increase the likelihood of disease activity [125]. Because the initial higher costs of HETs impact the short-term budgets of payers, the long-term indirect and societal benefits (e.g., improved productivity) of curbing disability accrual early may be considered beyond the remit of payers’ budgets [10]. Long-term cost-savings could therefore be lost in favor of short-term budget needs. Policy development from governmental and private insurers could ease these restrictions but is hindered by a lack of consensus from the MS community regarding optimal treatment and concerns about rising DMT costs [49, 126]. Results from the ongoing DELIVER-MS and TREAT-MS trials [18, 19] should be able to inform the development of national consensus statements and treatment algorithms necessary for policy reform. Concurrently, effective communication must be established with legislative decision makers and insurers to discuss DMT safety profiles and the clinical, financial, and societal consequences of poor disease control [10].

In addition to changes in insurance coverage, patient access to treatment in the US is impacted by unaffordable co-pays and out-of-pocket expenses from Medicare and commercial benefit designs [126–128]. A NARCOMS Registry survey exploring the effects of health insurance on DMT usage in MS revealed that 6.1% of patients with MS and specifically 9.2% with RRMS were unable to take DMTs due to insurance or financial reasons [126]. Overall, 7.8% of patients who obtained DMTs through insurance faced at least one insurance challenge, such as initial denial of their DMT use [126]. Personal finance considerations and challenges with co-pays influenced DMT usage, with approximately 25% of patients partially or completely relying on support from free or discounted drug programs [126]. The entry of more affordable generic and biosimilar medications into the market (such as those for dimethyl fumarate, fingolimod, teriflunomide, and natalizumab) attempts to counterbalance the expensive prescription drug market, but HET access is still restricted due to the stepwise approach of many insurance policies [129]. For patients covered by Medicare, generics do not necessarily offer budgetary relief as generic manufacturers are not required to provide discounts in the coverage gap, which normally contribute to a person’s total out-of-pocket spending [130].

There is clear need to alleviate the burden on patients trying to access appropriate MS care. Insurance company policies on DMT coverage that allow patients early access to the right treatment for them would provide substantial relief [124]. Pharmaceutical companies could be proactive in identifying patients with financial barriers and enroll them into patient assistance programs to help with drug costs and co-pays. Collaboration among neurologists, MS organizations, and patient advocacy groups may help to ensure fair access to DMTs; indeed, patients are already campaigning to address these issues [124, 131]. Involving regulatory authorities and health technology assessors could also help to facilitate cooperation from insurance companies [7].

In addition to prohibitive therapy costs, access to timely MRIs during the diagnostic and surveillance stages is further limited by out-of-pocket costs for the patient; the national average cost for an MRI in the US is $1325, which rises to $2250 in a hospital setting [132]. Moreover, variability and poor standardization in MRI protocols, machine and image quality, radiologist expertise, and personnel training in clinical practice can result in an inaccurate or incomplete MRI report that can delay critical treatment decisions [128]. Imaging centers should refer to the 2021 MAGNIMS-CMSC-NAIMS consensus recommendations on the use of MRI in patients with MS to standardize scanning protocols in clinical practice [133]. The Multiple Sclerosis Association of America (MSAA) has an MRI Access Program to assist with the cost of cranial and cervical spine MRI scans for people who have MS or are being diagnosed [134]. Routine MRI scans are instrumental in timely identification of early MS disease activity and therefore should be adequately covered to allow for rapid and informed treatment decisions made by HCPs and patients.

Prescriptive insurance policies undermine the therapeutic alliance between patients and HCPs and can lead to treatment failure and delays in appropriate care, resulting in poor adherence, patient anxiety, and worsening MS disease activity and disability [124, 127]. As an alternative, early and unrestricted access to HETs and diagnostic and monitoring tests can provide freedom of choice for clinicians, optimize outcomes for patients, and potentially reduce payer and societal costs [10]. The latter approach is in line with a therapeutic strategy that recommends making all DMTs, including HETs, available to people with relapsing forms of MS to identify the optimal treatment for each person based on perceived efficacy or tolerability rather than cost [7]. Until this becomes the norm, patients and clinicians may have to spend considerable time petitioning insurance companies to obtain the appropriate medication [124, 135].

### HCP- and service-related factors

The importance of preserving neurological function in people with MS by reducing delays at all stages of the care pipeline has been emphasized in a policy report by international MS experts [7]. However, delays commonly occur between MS symptom onset and confirmatory diagnosis by a specialist neurologist, arising due to administrative issues and poor awareness of MS symptoms among HCPs, patients, and their families [7]. National bodies, patient advocacy groups/associations, and/or professional bodies should aim to educate primary care physicians on referring suspected cases of MS promptly to a neurologist (preferably an MS specialist) to expedite appropriate care [7]. Referral delays may be exacerbated by neurologist hesitancy caused in part by the limited clinical guidance on early HET use and safety concerns [7, 8]. Familiarity with established DMTs is an influential factor and can result in treatment inertia (reported in up to 70% of neurologists) and delays switching from an established to a newer therapy, especially among non-MS neurologists [127, 136]. In the US, HCPs might be even more risk averse than patients due to different perspectives and clinical experience regarding potentially life-threatening safety risks, which can encourage more defensive medical practices out of concern for legal liability [137, 138]. Updated long-term safety profiles, which can be communicated through continuing medical education and peer-to-peer events [10, 139], will be critical for managing perceptions of HCPs and non-specialist neurologists in this regard.

Another reason for HCP hesitancy around early HET is the variance in vaccine response highlighted in the wake of the coronavirus disease 2019 (COVID-19) pandemic. The ability of anti-CD20 HETs to deplete B lymphocytes and potentially cause hypogammaglobulinemia raised concerns about whether HET-treated patients could achieve the appropriate immune response to a COVID-19 vaccine [3, 140]. Ocrelizumab has even been associated with a more severe course of COVID-19 in a real-world analysis [141]. However, there have been several reports of patients receiving anti-CD20s for MS being able to mount robust antigen-specific CD4 + and CD8 + T-cell responses to messenger RNA COVID-19 vaccines, even with impaired humoral responses [142–145]. Moreover, the DMT risks associated with COVID-19 may have been overestimated in earlier studies due to confounders [146], and HET effects on COVID-19 severity or infection risk vary according to patient population and treatment [147, 148]. Improving education about vaccine efficacy and the benefit-risk ratio of HETs regarding infection risk can help to address HCP concerns. A multidisciplinary approach to early treatment, involving MS specialist nurse navigators and pharmacists serving as accessible resources, can support the shared decision-making process and achieve more well-informed patient care [149, 150]. There may also be a call for involving infectious disease experts to manage timely risk mitigation in vulnerable patients.

For HCPs, selecting among many different DMTs can become even more challenging with time and financial constraints in clinical settings [127]. MS specialists must have sufficient time in the clinic to adequately educate patients on early treatment options [7]. Improving the quality and efficiency of decision-making in clinical practice can be assisted by comprehensive treatment algorithms using prognostic factors to identify higher-risk patients who would benefit the most from early HET [24, 151]. The eventual goal would be a personalized treatment approach encompassing prognostic factors (clinical, paraclinical, environmental, and demographic), patient-related factors (e.g., comorbidities, family planning, and level of risk aversion), and drug-related factors (i.e., safety, cost, and treatment sequencing options) [24]. Involving specialty pharmacists in the medication management of patients with MS may also relieve some pressure while optimizing patient care [152].

Additional systemic factors, such as poor continuity of care outside of MS clinical centers, long wait times to access secondary care, and inherent limitations in the ability of conventional MRIs to capture cortical lesions and regional atrophy, create problems for obtaining appropriate health care [150, 153, 154]. There may also be practicable challenges, as most HETs (alemtuzumab, natalizumab, ocrelizumab, and ublituximab) require intravenous administration at specialized infusion centers or tertiary hospitals [26, 29, 155]. Ofatumumab is an exception and can be self-administered by subcutaneous injection without the need for an infusion [156]. Access to infusion sites can have the additional benefit of promoting adherence [155]. An international panel of MS specialists and multidisciplinary reviewers recommend offering infusible therapy within 4 weeks following the patient’s agreement to initiate treatment, with an aspirational goal of initiating the DMT within 7 days [157]. To achieve this, resources at infusion sites should be strategically increased and/or expanded to additional locations and times to alleviate resource demand [155]. Expanding access to laboratory and vaccination centers would also expedite necessary screening and vaccinations prior to starting DMT.

Overcoming issues of hesitancy regarding early HET could be largely ameliorated by a consensus statement from national neurological and MS associations in the US. With this in place, expanding the future MS workforce by providing more educational and research grants in neurology and neuroimmunology could help to ensure that receptivity to the early HET approach is sustained over the long-term.

### Patient-related factors

Different experiences in patients’ engagement with health care resources can influence their treatment decisions. First, health care resource utilization is lower among patients from underserved populations who are at high risk for disability, such as those from lower socioeconomic and minority racial/ethnic backgrounds [158–161]. Inequalities in access to health care services for people with MS are worse for men, older patients, lower socioeconomic groups/least educated, non-White (including African American and Hispanic), those with mental health problems, and those residing in rural areas [162–164]. People with MS who can see neurologists are more likely to receive DMTs and access specialists than those who see other providers, but the probability of seeing a neurologist is significantly lower for people who do not have health insurance, are poor, are living in rural areas, are African American/Hispanic, or have worse disability [158, 165, 166]. Access is further limited by the poor geographic distribution of MS centers across the US providing subspecialty care [166]. Both Black and Hispanic patients with MS frequently initiate low- and moderate-efficacy DMTs despite a greater risk of developing a severe disease course with greater disability [167, 168].

Second, even patients who are familiar with a neurologist or other HCP may not sufficiently learn about DMTs during routine consultations [169]. For example, studies have found that patients strongly favored medications that could improve symptoms, despite this not being the primary effect of DMTs [169]. Some patients have reported difficulties in finding relevant information about DMTs on the internet that does not require a high education level [170]. Comorbidities such as MS-related cognitive impairment, depression, fatigue, or anxiety may then contribute to poor understanding [169]. Furthermore, maintaining adherence can be a challenge for most MS medications, with reported rates of DMT adherence ranging from 52 to 92.8% [171]. Poor adherence has been linked to worse disease outcomes and increased costs [118] and may make an effective DMT appear ineffective, leading to unnecessary therapy changes [128].

Patients could be empowered to make fully informed decisions on their initial DMT by increasing access to MS centers and improving the quality of HCP-patient interactions [7, 127, 150]. A tailored, multifaceted approach to DMT selection may help to identify patients, especially those in underserved populations, who would most benefit from HET to maximize their treatment outcomes [168]. Low-income minorities can be encouraged to initiate and adhere to treatment through adequate education about the disease course, treatment goals and options, and community resources [159]. The MSAA and National Multiple Sclerosis Society provide information and resources in Spanish to serve the rapidly growing US Hispanic population [172, 173]. In response to inequalities in MS care among underserved populations, ongoing US-based registries for Hispanic and African American populations with MS have been established to collect incidence and prevalence data, educate patients and HCPs, and learn about issues affecting access to MS care [164, 174].

HCPs have a responsibility to support patients in understanding their options during clinical discussions. They should demystify important topics, such as the benefits of early treatment; information on new DMTs, without overemphasizing the perceived risks; and the consequences of suboptimal treatment [7, 127]. Counseling patients on DMT use around pregnancy may also support early HET use in those who feel inadequately informed about the implications on family planning [175, 176]. Patient engagement could benefit from the use of tools, such as decision aids, health coaching, and question prompts, along with providing personalized information, using motivational interviewing, and directing to useful resources [177]. Reliable sources of information on HETs include patient advocacy websites, such as the MSAA Ultimate MS Treatment Guide [178], MS neurologists’ curated platforms on social media [179, 180], and the MS Living Well website with its associated podcast [181], which educate hundreds of thousands of patients globally.

Shared decision-making between HCPs and patients is a key component to improving acceptance of, and adherence to, DMTs [56, 127]. It also allows patients to communicate their preferences, which may differ from those of HCPs [137, 138, 169]. For instance, route of administration is an important consideration for many patients [182]. Likewise, patient concerns about the long-term risks of early intervention with HETs, particularly infections and malignancy [8], may motivate preferences for DMTs with a perceived lower risk of significant side effects [169]. Others may prefer treatments with only moderate, but guaranteed benefits [169]. American Academy of Neurology guidelines recommend that HCPs take into consideration patient preferences around safety, administration route, medication frequency, monitoring, and lifestyle when deciding on an initial therapy [56]. Certain HETs could satisfy such patient requirements: both ocrelizumab (administered twice a year by intravenous infusion) and ofatumumab (self-administered once a month by subcutaneous injection) have demonstrated superior adherence and persistence to other non-HET injectable and oral DMTs [183–185]. Sharing all therapeutic options with patients, including those related to an early HET approach, encourages greater participation in their health care management—an outcome that could not only improve disease education and treatment satisfaction but also ultimately improve their long-term health outcomes.

## Conclusions

Emerging evidence supports initiating early HET after an MS diagnosis to maximize patient outcomes. Implementing early HET in the US is hindered by various barriers that delay much-needed updates to best practices. Encouraging adoption of early HET strategies will be aided by long-term safety data to update clinical guidelines and mitigate safety concerns, improving patient and HCP education to empower shared decision-making for all patients regardless of background, and implementing policy changes that expand access to HETs at the local and national levels. Advocacy is needed at all levels—HCPs, insurers, and patient groups—to encourage reevaluation of current clinical guidelines and approval of first-line HET on insurance formularies. Doing so may be imperative to creating meaningful change in the US health care system and continually improving outcomes for people with MS.

## Data Availability

Not applicable.
